# Advancing cluster randomised trials in children’s therapy: a survey of the acceptability of trial behaviours to therapists and parents

**DOI:** 10.1186/s13063-022-06872-y

**Published:** 2022-11-26

**Authors:** Samantha Armitage, Tim Rapley, Lindsay Pennington, Jennifer McAnuff, Elaine McColl, Catherine Duff, Rob Brooks, Niina Kolehmainen

**Affiliations:** 1 Sheffield Children’s NHS Foundation Trust, Sheffield, UK; 2 Northumbria University, Newcastle upon Tyne, UK; 3 Newcastle University, Newcastle upon Tyne, UK; 4 Newcastle upon Tyne NHS Hospitals Trust, Newcastle, UK; 5 Leeds Community Healthcare NHS Trust, Leeds, UK; 6 University of Bradford, Bradford, UK

**Keywords:** Cluster randomised controlled trials, Trial design, Child, Physiotherapy, Occupational therapy, Speech and language therapy

## Abstract

**Background:**

Randomised controlled trials of non-pharmacological interventions in children’s therapy are rare. This is, in part, due to the challenges of the acceptability of common trial designs to therapists and service users. This study investigated the acceptability of participation in cluster randomised controlled trials to therapists and service users.

**Methods:**

A national electronic survey of UK occupational therapists, physiotherapists, speech and language therapists, service managers, and parents of children who use their services. Participants were recruited by NHS Trusts sharing a link to an online questionnaire with children’s therapists in their Trust and with parents via Trust social media channels. National professional and parent networks also recruited to the survey. We aimed for a sample size of 325 therapists, 30 service managers, and 60 parents. Trial participation was operationalised as three behaviours undertaken by both therapists and parents: agreeing to take part in a trial, discussing a trial, and sharing information with a research team. Acceptability of the behaviours was measured using an online questionnaire based on the Theoretical Framework of Acceptability constructs: affective attitude, self-efficacy, and burden. The general acceptability of trials was measured using the acceptability constructs of intervention coherence and perceived effectiveness. Data were collected from June to September 2020. Numerical data were analysed using descriptive statistics and textual data by descriptive summary.

**Results:**

A total of 345 survey responses were recorded. Following exclusions, 249 therapists and 40 parents provided data which was 69.6% (289/415) of the target sample size. It was not possible to track the number of people invited to take the survey nor those who viewed, but did not complete, the online questionnaire for calculation of response rates. A completion rate (participants who completed the last page of the survey divided by the participants who completed the first, mandatory, page of the survey) of 42.9% was achieved. Of the three specified trial behaviours, 140/249 (56.2%) therapists were least confident about agreeing to take part in a trial. Therapists (135/249, 52.6%) reported some confidence they could discuss a trial with a parent and child at an appointment. One hundred twenty of 249 (48.2%) therapists reported confidence in sharing information with a research team through questionnaires and interviews or sharing routine health data. Therapists (140/249, 56.2%) felt that taking part in the trial would take a lot of effort and resources. Support and resources, confidence with intervention allocation, and sense of control and professional autonomy over clinical practice were factors that positively affected the acceptability of trials. Of the 40 parents, twelve provided complete data. Most parents (18/40, 45%) agreed that it was clear how trials improve children’s therapies and outcomes and that a cluster randomised trial made sense to them in their therapy situation (12/29, 30%).

**Conclusions:**

Using trials to evaluate therapy interventions is, in principle, acceptable to therapists, but their willingness to participate in trials is variable. The willingness to participate may be particularly influenced by their views related to the burden associated with trials, intervention allocation, and professional autonomy.

## Background

Formal, randomised controlled trial designs are important for robust evaluation of the effectiveness of health interventions [1, 2]. By assessing causal relationships between an intervention and outcomes, trials provide valuable information about potential intervention benefits to inform decision-making about finite healthcare resources. However, randomised controlled trials (RCTs) have not been equally adopted and used in all health fields and populations. Whilst they are well-established in many acute adult health conditions and increasingly used in paediatric medicine, trials remain relatively rare in children’s therapy interventions including occupational therapy, physiotherapy, and speech and language therapy. A scoping of the International Standard Randomised Controlled Trials Number (ISRCTN) registry in October 2020 (unpublished observations) found 1892 studies registered with child participants globally, of which only twelve were open, ongoing trials investigating childhood non-pharmacological interventions in therapy contexts [3–14].

We, and others [15, 16] in the field of children’s therapy interventions, argue that establishing the effectiveness of interventions is central to high-quality care, to optimal achievement of health outcomes, and to decision-making and that advancing robust evaluations of non-pharmacological interventions is an important research priority. However, others have argued that successful adoption of feasible and acceptable trial designs to evaluate children’s therapy is very difficult, if not impossible [17–20]. Key arguments against trials have included that some of the fundamental features of RCTs, such as the delineation and control of variation, and the focus on a restricted number of specified outcomes [18, 19] are not compatible with the fundamental ideas of children’s therapy where variation equates to important context to be incorporated into interventions, and outcomes are commonly longitudinal, multi-level, and interwoven with the experience of therapy [17, 19, 21, 22]. Yet, there are published examples of successful trials of therapy interventions in general [23–26] and with children specifically [27–29], particularly using cluster randomised controlled trial designs [25, 27, 29] which suggests that trials are feasible and acceptable, at least with some interventions, some of the time.

The present paper proposes that at least some types of RCTs, particularly cluster RCTs, may be feasible in children’s therapy and that trials offer a potentially under-used approach to advancing children’s non-pharmacological interventions and child health. It further investigates some key questions that remain to be answered before progress at scale is possible, specifically: What are the potential barriers to using large-scale formal randomised controlled trials to evaluate children’s therapies in clinical practice? What are the views among therapy professionals and service users about behaviours required for their involvement in the trials? Where are the congruences and divergencies in the professionals’ and service users’ attitudes?

The specific aim of our study was to investigate the views of allied health therapists, as the most common paediatric non-pharmacological intervention providers, and parents of children receiving these services, about the acceptability and compatibility of RCTs in the context of paediatric therapy. This was to generate evidence to inform the design of future trials of paediatric non-pharmacological interventions.

## Method

This was a national survey of United Kingdom (UK) paediatric allied health therapists (occupational therapists, physiotherapists, speech, and language therapists), and parents of children who use their services, about cluster randomised controlled trials (CRCTs) in children’s therapy. In CRCTs, clusters of some kind (e.g. therapy teams, children’s centres, people living in certain areas) are randomised to the study arms, and data within a cluster is considered connected rather than independent. CRCTs are used in situations where it is preferable to assign groups of people to an intervention, rather than individuals [30], and may go some way to address some of the difficulties with trials in children’s therapy. CRCTs have been successfully employed in adult non-pharmacological intervention contexts [25, 31, 32] and are generally considered a potential way forward in children’s therapy trials.

The present study was approved by the National Health Service (NHS) Health Research Authority (HRA) and North West - Greater Manchester West Research Ethics Committee (REC, ref: (19/NW/0521). The survey is reported here in accordance with the Checklist for Reporting Results of Internet E-Surveys (CHERRIES) [33].

Therapists and therapy service managers were sampled from a database of children’s therapy services in the UK [34]. The research departments of 122/149 NHS Trusts/Health Boards named on the database and specifically based in England and Wales were approached either directly by the research team or via the National Institute of Health Research (NIHR) Clinical Research Network (CRN). Therapists and managers in children’s therapy services were then approached and invited to the study by their local research teams. The total number of individual therapists and service managers in every therapy service approached is unknown. Professional networks with UK-wide membership also invited their members to participate, specifically the Royal College of Occupational Therapists Specialist Section for Children, Young People and Families, the Royal College of Occupational Therapists Specialist Section for Housing (social care), the Association of Paediatric Chartered Physiotherapists, and the Royal College of Speech and Language Therapists Clinical Excellence Networks. Due to data protection regulations, no member details were shared with the research team and the professional network organisations distributed the survey to their own members. The total number of individuals receiving the survey is therefore unknown along with the degree of duplication between sampling sources, i.e. the number of individuals that received the survey from their NHS Trust and professional networks. Parents of children were invited to the study via participating NHS Trusts’ Facebook and Twitter social media pages, the South and West Yorkshire Children’s Additional Need Networks, the Northern Ireland Cerebral Palsy Register, and regional Parent Carer Forum networks. Communication Matters magazine, Augmentative and Alternative Communication (AAC) Info newsletter, and Sensory Integration Network UK and Ireland with a shared therapist and parent membership also supplemented recruitment. HRA- and REC-approved invitations to participate in the study, including an open link to the online participant information sheet and questionnaire, were emailed to potential participants by participating organisations or advertised on organisations’ social media and/or online newsletters. Participant information sheets provided information for informed consent. No personal, identifiable information was collected, and participants were informed of data management processes as part of the informed consent process. Consent was inferred from questionnaire completion.

We aimed to achieve a sample size of 30 managers of therapy services from across the UK, 325 therapists providing care to children with disabilities, and 60 parents of children receiving therapy. Sample sizes were based on the information available in the sampling frame of children’s therapy services [34] and on anticipated response rates of 20% for managers and therapists and 5% for parents. These expected response rates were informed by response rates achieved in previous national surveys of children’s therapy services [35].

An online questionnaire was developed to collect data on the acceptability of cluster randomised controlled trials in the context of children’s therapy. The questionnaire was structured with three questions about the general acceptability of trials at the beginning, followed by vignettes to specify each of the trial behaviours and acceptability questions based on the vignettes, and finally a section on participant demographics at the end of the questionnaire. Documents related to the development of the questionnaire and the final questionnaires for therapists and parents can be accessed at 10.25405/data.ncl.17087333.v1.

Trials were specified by therapist and parent participant behaviours, and the behaviours were specified in terms of target, actions, context, time (TACT [36]; see Tables 1 and 2). Three behaviours for therapists and three behaviours for parents were incorporated into vignettes for an online questionnaire. Question items and response options related to the vignettes were developed to investigate the acceptability of the behaviours.

**Table 1:**
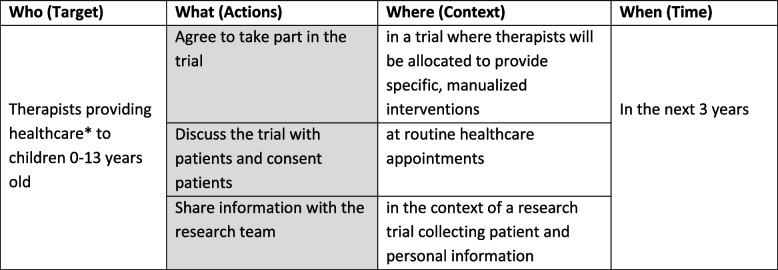
Target behaviours related to therapists specified by TACT

Grey box = the main aspect on which the vignette is seeking to elicit views

^*^Any type of healthcare, including assessment, intervention, and advice

**Table 2:**
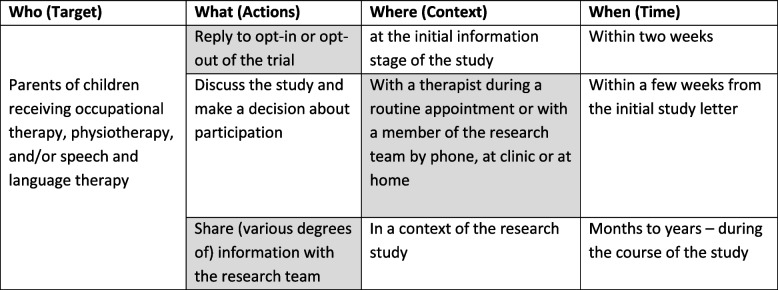
Target behaviours related to parents specified by TACT

Grey box = the main aspect of the behaviour on which the vignette is seeking to elicit views

The theoretical framework of acceptability [37] (TFA) provides an explicit definition of acceptability: ‘A multi-faceted construct that reflects the extent to which people delivering or receiving a healthcare intervention consider it to be appropriate, based on anticipated or experiential cognitive and emotional responses to the intervention’ [37] and proposes 7 constructs relating to acceptability:

1. Affective attitude (how an individual feels about the intervention)
2. Burden (perceived amount of effort to participate in the intervention)
3. Ethicality (the extent to which the intervention has a good fit with an individual’s value system)
4. Intervention coherence (the extent to which the individual understands the intervention and how it works)
5. Opportunity costs (the extent to which benefits, profits or values must be given up to engage in the intervention)
6. Perceived effectiveness (the extent to which the intervention is perceived as likely to achieve its purpose)
7. Self-efficacy (the individual’s confidence that they can perform the behaviour(s) required to participate in the intervention)

Constructs from the TFA [37, 38] underpinned the survey questions and response options, with modifications. The TFA focuses on *acceptability of an object* (i.e. an intervention), whereas our study focused on the *acceptability of behaviours* related to an object (i.e. actions related to participating in a trial). We used the TFA to consider prospective (rather than present) acceptability as a predictor of future behaviour. Further theoretical constructs were incorporated into the acceptability framework to reflect the specific study context; for example, literature on behavioural regulation [39] and the Theoretical Domains Framework [40] were used to map the construct of ethicality to ‘goals’ and the value systems inherent in goals, in addition to the TFA construct ‘fairness’ [38].

Question items related to trial behaviours (Tables 1 and 2) were developed for the acceptability constructs: confidence (self-efficacy), burden and affective attitude, with a mix of open and closed response options. Likert scales, drawn from the theory of planned behaviour [41], were used in closed response options. Question items were then developed to measure the general acceptability of trials for the acceptability constructs of intervention coherence and perceived effectiveness. Draft questionnaires were reviewed within the research team and piloted with health professionals (2 health visitors and 1 occupational therapist) and parents (1 mother and 1 father). Piloting involved cognitive testing [42] to check the construct validity and think-aloud techniques [42] to collect feedback on the formatting of the questionnaire. In response to piloting, changes were made to the questionnaire (see 10.25405/data.ncl.17087333.v1). The final questionnaire was hosted and administered online by Qualtrics [43]. Accessibility checks on the questionnaire were reviewed in Qualtrics and deemed to be fair. To improve accessibility, question items were presented to participants sequentially; display and skip logic were applied to navigate therapists and parents through the appropriate questionnaire; and page navigation buttons (back and next) were made available for participants to review their answers and move forwards and backwards through the questionnaire. The first survey question, related to the general acceptability of trials, was set as mandatory with all subsequent questions set for voluntary responses. No incentives were offered for questionnaire completion.

Data were transferred from Qualtrics to the Statistical Package for Social Sciences (SPSS) v25 [44] for analysis. Descriptive statistical analysis was used to summarise the numerical data from closed-question responses. Missing data were excluded from the analysis of each questionnaire item. Questionnaire data were primarily ordinal (Likert scales), resulting in modal and median values being used for measures of central tendency, and interquartile range and range being used to describe dispersion. For the two continuous variables related to participants’ demographic data (number of years the participating therapist has been qualified and the age of the participating parents’ child), the null hypothesis that the data followed the normal distribution was rejected based on the Shapiro-Wilk test and visual inspection of the distribution of values. The median, interquartile range (IQR), and range were therefore used to summarise the continuous variable data. Comments written in open-text response boxes were extracted from the questionnaires and pooled into two categories (likes and dislikes) and read and summarised descriptively into factors related to participants’ positive and negative attitudes towards trials.

## Results

**Fig. 1:**
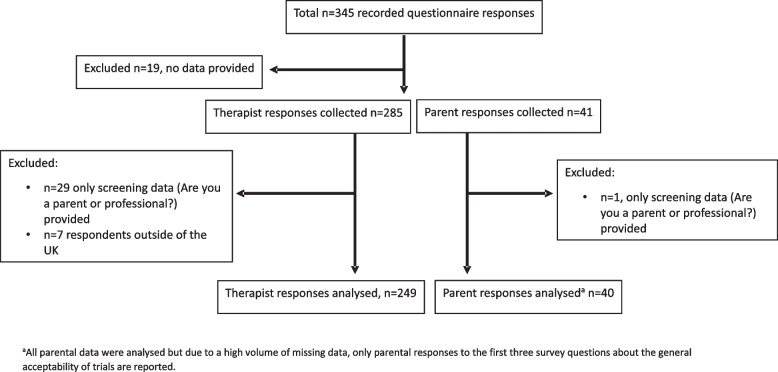
Flow chart of survey participant eligibility

The survey was open to collect data between June and September 2020. The total number of people—professionals and parents alike—who received the invitation email and visited the survey site is unknown, precluding the calculation of survey response rates. A total of 345 survey responses were received, of which 289 were eligible for inclusion in the final analysis. Figure 1 presents a flow chart of survey questionnaire responses by respondent group (therapist/parent) and inclusion eligibility. We achieved an overall completion rate, based on users who completed the last page of the survey divided by the number who completed the first (mandatory) page of the survey [33], of 42.9%. The potential for duplicate responses could not be determined.

In total, 249 therapists and 40 parents recorded survey responses on trial behaviours. Characteristics of therapist participants are described in Table 3; not all respondents completed these demographic and work-related variables due to the number of incomplete questionnaires and collection of demographic data at the end of the questionnaire. The median number of years qualified for 116/249 (46.6%) therapists reporting was 17 (IQR: 6–25 range 0–38).

**Table 3:** Characteristics of therapist participants

Characteristic (number of responses)	Therapist/service manager responses, ***N*** (%)
Location of employing organisation (117/249, 47%)	
England	112/117 (95.7%)
Wales	3/117 (2.6%)
Scotland	2/117 (1.7%)
Northern Ireland	0/117 (0%)
Profession (115/249, 46.2%)	
Occupational therapy	48/115 (41.7%)
Speech and language therapy	38/115 (33.0%)
Physiotherapy	29/115 (25.2%)
Professional role (116/249, 46.6%)	
Therapist	99/116 (85.3%)
Service manager	9/116 (7.8%)
Others, described as follows:	8/116 (6.9%)
Clinical lead	
Lead OT: clinical work + leadership role	
Principal paediatric OT schools, split of both therapist and manager	
Team leader	
Team manager/therapist	
Care setting (110/249, 44.2%)	
NHS	100/110 (90.9%)
Independent practice	5/110 (4.5%)
Local authority/council	3/110 (2.7%)
Others, described as follows:	2/110 (1.8%)
Education	
Government department	

Fourteen (*n* = 14/40, 35%) of the responding parents provided sociodemographic data which is presented in Table 4. The median age of the parents’ children was 6 years (IQR: 5–10.5, range 3–14). Twelve of the fourteen (85.7%) parents reported their child had diagnosed health conditions (*n* = 6/12, 50% autism spectrum disorder/Asperger’s syndrome/specific language impairment; *n* = 5/12, 41.7% ‘other’ described as anxiety and demand avoidance, a rare genetic condition, Down syndrome, sensorial processing disorder and hypersensitivity; *n* = 4/12, 33.3% developmental coordination disorder/dyspraxia/verbal dyspraxia; *n* = 4/12, 33.3% developmental delay/speech delay; *n* = 4/12, 33.3% cognitive delay/learning disability; *n* = 2/12, 16.7% cerebral palsy). The median number of health conditions children were reported to have was 2 (IQR: 1–3, range 1–5).

**Table 4:** Characteristics of parent participants

Characteristic (number of responses)	Parent responses, ***N*** (%)
Gender (14/40, 35%)	
Female	12/14 (85.7%)
Male	2/14 (14.3%)
Language(s) spoken at home (14/40, 35%)	
English	14/14 (100%)
Others, described as follows:	1/14 (7.1%)
Greek	
Child’s main carer (14/40, 35%)	
Mother	14/14 (100%)
Father	0/14 (0%)
Main carer’s highest qualification (14/40, 35%)	
University doctorate	0/14 (0%)
University master’s degree	2/14 (14.3%)
University bachelor’s degree	7/14 (50%)
College-level qualification (HSC/A level)	1/14 (7.1%)
Secondary education certificate (O-level, GCE, GCSE)	3/14 (21.4%)
Others, described as follows:	1/14 (7.1%)
Currently in university	

**Fig. 2:**
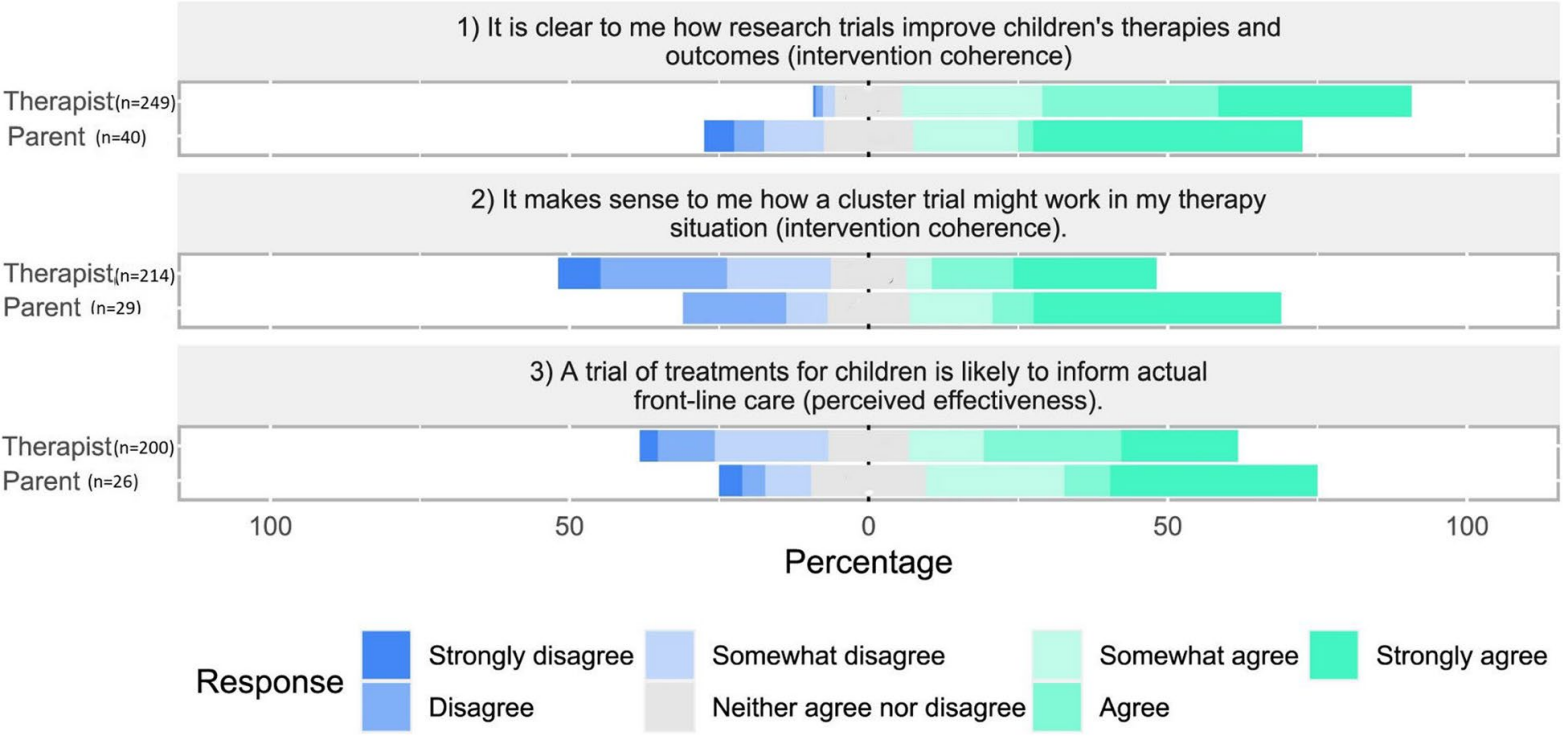
General acceptability of cluster trials to therapists and parents of children receiving therapy

Of the 40 parents who participated in the survey, 26/40 (65%) ceased answering after the first three survey questions related to the general acceptability of trials. Fourteen of the 40 (35%) parents continued the questionnaire but only 12/40 (30%) answered all questions. Due to the large amount of missing data, we report parental data on the first three questions related to the general acceptability of trials only. Figure 2 presents the general acceptability of trials to children’s therapists and parents of children receiving therapy respectively.

**Fig. 3:**
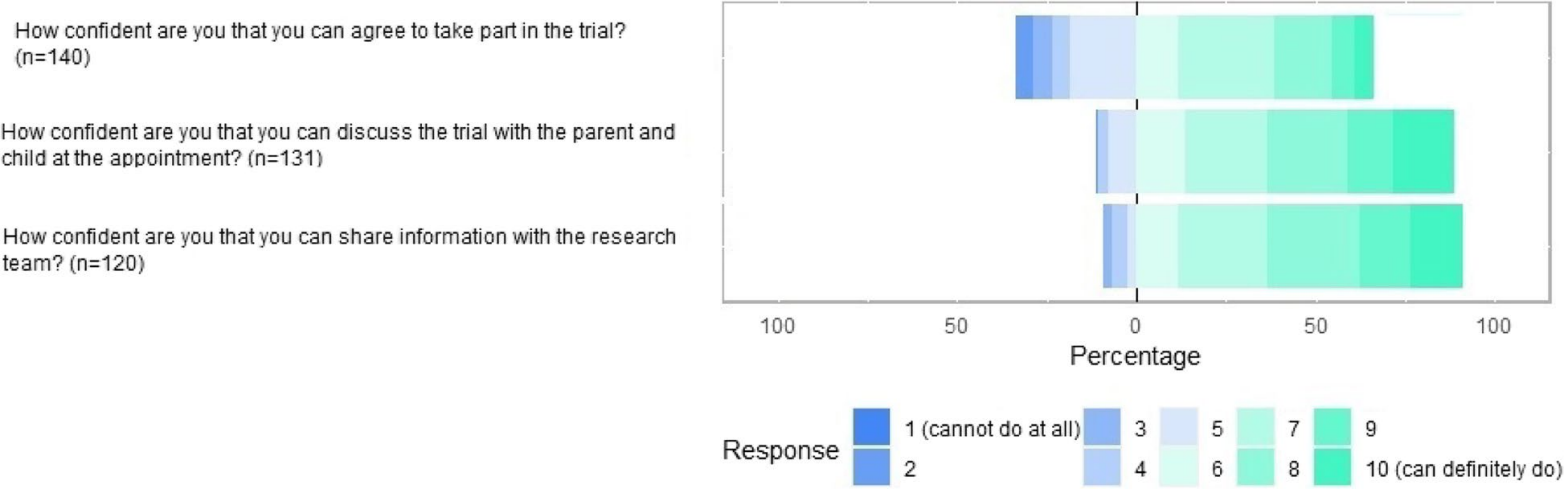
Therapists’ confidence (self-efficacy) in each of the specified trial behaviours

**Fig. 4:**
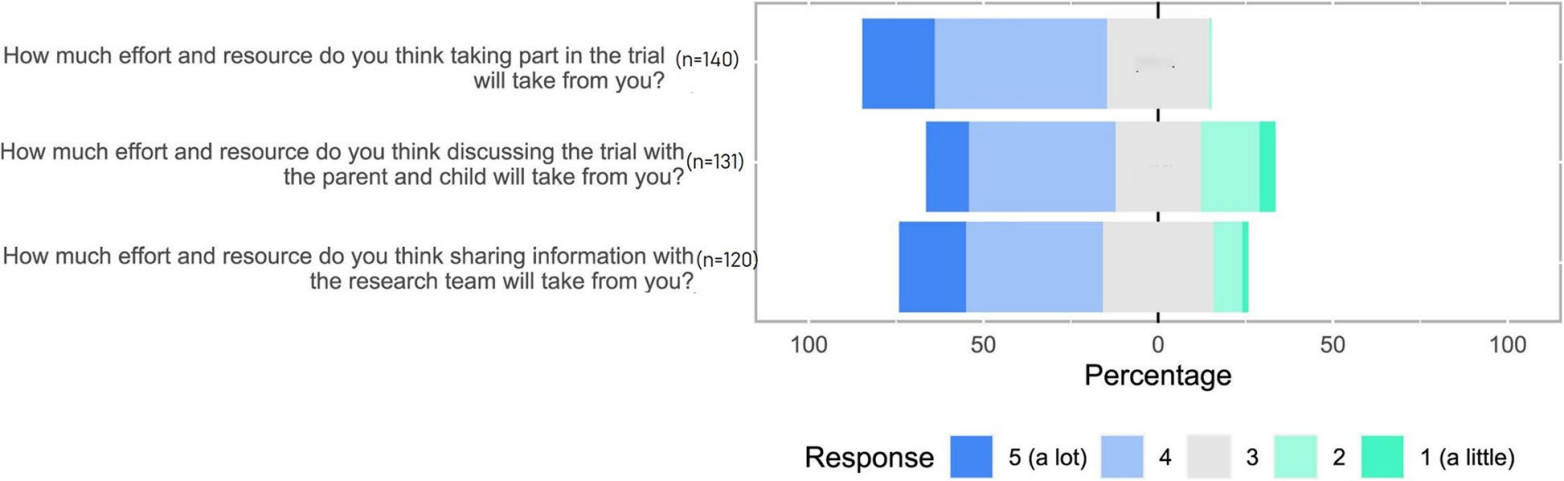
Therapists’ perceived effort and resource (burden) for each of the specified trial behaviours

Most commonly, therapists and parents agreed that it was clear to them how trials improve children’s therapies and outcomes (TFA construct ‘intervention coherence’). Parents (*n* = 18/40, 45%) tended to strongly agree with this statement more than therapists (*n* = 81/249, 32.5%) but had more varied views (see Fig. 2). Therapists were somewhat divided that it made sense to them how a cluster trial would work in their therapy situation (TFA construct ‘intervention coherence’) and that a trial of treatments for children would likely inform front line care (TFA construct ‘perceived effectiveness’) whereas parents tended to show more agreement with these statements than therapists (see Fig. 2). The acceptability of specified trial behaviours to children’s therapists are presented in Figs. 3 and 4, grouped by TFA acceptability constructs confidence (self-efficacy) and burden [37].

Therapists’ confidence that they could take part in a trial was varied (see Fig. 3). Only 8/140 (5.7%) of the reporting therapists felt confident that taking part in the trial was something they could definitely do. Mostly, therapists felt somewhat confident they could take part in the trial. Therapists were more confident that, if taking part in the trial, they could discuss the trial with the parent and child at an appointment and share information with the research team, either directly through questionnaires and interviews or indirectly through sharing routine health data (see Fig. 3).

Across all three trial behaviours, therapists perceived that ‘moderate’ to ‘a lot’ of effort and resource would be required from them and tended to perceive the trial behaviours as burdensome (see Fig. 4). Taking part in the trial tended to be perceived as the most burdensome behaviour. Therapists’ perceptions of effort and resource were most widely dispersed in relation to the behaviour ‘discussing the trial with the child and parent at the appointment’ where some therapists perceived this could be done with little effort and resource (see Fig. 4).

**Fig. 5:**
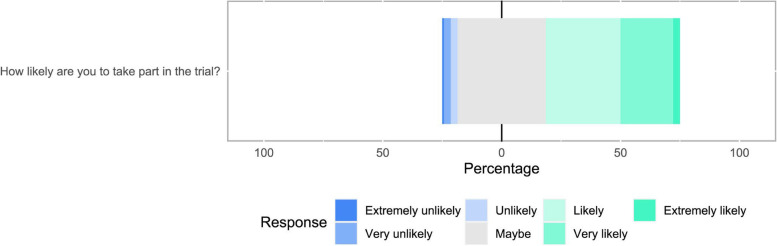
Likelihood of therapists taking part in a trial

Figure 5 represents therapists’ (*n* = 140/249, 56.2%) intentions to take part in a trial. Therapists were uncertain that they would agree to take part in a trial, most commonly (*n* = 52/140, 37.1%) rating their intention to take part in a trial as ‘maybe’.

Commonly, across all three trial behaviours (Table 1), therapists reported positive feelings (TFA construct ‘affective attitude’) in relation to (i) receiving support for the trial to be put in place, (ii) the learning and development that they would gain from participating in a trial, (iii) children being represented in the trial, and (iv) therapist and parent choice and control over if and how much they are involved in the trial (see Table  5).

**Table 5:** Participant identified factors related to positive affective attitude about trials

Factors related to positive affective attitude	Sample verbatim text responses from therapist participants
i) Receiving support for the trial to be put in place	• Support given by the team • Support that will be given by the researchers • Management support • Support from the research team and my employer
ii) The learning and development that they would gain from participating in a trial	• Training on how to provide [intervention] is a professional development • Learning and CPD [Continuing Professional Development] opportunities • Training given on how to approach this conversation • Learning more about the research process through training and active involvement
iii) Children being represented in the trial	• Useful to gather information to represent children’s experiences • Important to know about the children’s health, difficulties, and development as working with very complex children • Background information is vital for creating results that reflect the participants (varying abilities, difficulties, circumstances, protected characteristics, etc.)
iv) Therapist and parent choice and control over if and how much they are involved in the trial	• Well explained to parents and gives them a choice • Parental choice to take part • Respects parent choice • It is clear the parent can choose not to be involved • The parent has the ultimate choice • Lots of choices • Level of choice • Information to be shared is up to me and in my control • Being able to choose information shared • Still have the power to say what ok/not ok with • The fact that I would have choice and autonomy over what info is shared

Negative feelings (TFA ‘affective attitude’) were similarly reported across all three behaviours and related to (i) time, therapist capacity, and resources needed to participate in a trial; (ii) confidence in the new intervention; (iii) (dis)agreement with the benefit of intervention allocation; and (iv) loss of autonomy (see Table 6).

**Table 6:** Participant-identified factors related to negative affective attitude about trials

Factors related to negative affective attitude	Sample verbatim text responses from therapist participants
i) Time, therapist capacity, and resources	• Concerned about time commitments on top of day job • Would this be in addition to my normal caseload or just replace some treatments for the eligible children? • Additional time required in order to provide additional intervention • Potential time implications • Potentially more work • Unclear how much extra time this will take – where time coming from – impact on other children on caseload
ii) Confidence in and with the new intervention	• Providing a ‘new treatment’ which the [therapist] does not have confidence in – or asking others to deliver – will impact on the effectiveness of the ‘treatment’. • Being randomly allocated without confidence that the treatment could work would not sit comfortably. • If the therapy was new and not already evidence based it might be difficult to agree to take part. • May not feel skilled/confident in the treatment • Not knowing the treatment/client group – I am more confident with some client groups than others. • My concern would be if the type of treatment being offered by the trial would not be what I would usually do and I would have concerns regarding the quality of care and outcomes.
iii) (Dis)agreement with the benefit of intervention allocation	• I might not agree with the treatment I am implementing, and I might feel bad that the child is not getting the usual interventions. • May be a treatment approach that I don’t agree with or feel may not be the most beneficial to the child • May still clash with professional judgement on what intervention would be most effective
iv) Loss of autonomy	• Loss of individuality for selection of appropriate treatments • Concerns about possible restrictions when creative problem-solving • That won’t have choice of treatments for a child I see • If after a while it became apparent that the therapy isn’t working/appropriate for the child I would be concerned about the lack of flexibility to change the approach.

## Discussion

Randomised controlled trials to evaluate children’s therapy have long been a topic of debate, focussed primarily on the incompatibility of trial design with children’s therapy interventions and practice [18–20]. Our study investigated the acceptability of selected trial behaviours, in the context of various trial design features, to children’s therapists and parents of children using therapy services with the aim of advancing trial design. Trials and their coherence with children’s therapy, outcomes, and front-line care were generally acceptable to both therapists and parents. Therapists reported varied levels of confidence in participating in a trial and high perceptions of burden associated with taking part in a trial and uncertainty about their intention to do so. Trial factors that positively and negatively affect therapists’ attitudes to trials have been identified.

Acceptability factors identified by therapists in our study, e.g. positive support for the trial; time, capacity, and resources; and confidence with intervention allocation reflect factors commonly reported by healthcare professionals in other clinical trial settings as barriers to trial participation and recruitment [45–49]. Whilst these barriers initially appear behavioural, there may also be structural barriers that explain these. For example, whilst therapy time is frequently costed into research trials and finance provided to the therapy service to recompense for that time, this may be of limited benefit where the challenges relate to the unavailability of a workforce to absorb the additional workload. Understanding the structural barriers to therapists’ participation in trials and identifying practical solutions to address the barriers is important to reduce the perceived burden of trial participation. Some of the factors identified in our present study, such as supporting further therapy capacity building and learning as part of the trial, offer new insights into avenues to enable a step change in children’s therapy services’ capacity to be involved in and deliver trials. Similarly, therapists’ positive feelings and confidence in taking part, and providing support and resources for implanting and embedding the trial in the practice setting, are likely very important.

The findings of our study influence the future direction of trials methods research and the use of trials in children’s non-pharmacological interventions. Key questions for future research have been identified and relate to (i) how different solutions and supports to address capacity and caseload pressures affect therapists’ involvement in trials; (ii) what strategies work best, what works for whom and in which contexts in terms of incentivising therapists’ trial participation by embedding meaningful support, learning, and development opportunities within trials; (iii) what strategies can be used to effectively address concerns about perceived choice, control, and professional autonomy within trials; and finally (iv) what strategies can be adopted to facilitate parent and child engagement in trials and what trial methods and processes work best for parents and children.

For our survey, we used a broad and comprehensive sampling strategy alongside proactive recruitment and an online survey, yet the final sample size was smaller than originally hoped for, and most parents did not complete the entire questionnaire, with insufficient responses to the vignette items for meaningful analysis. The COVID-19 pandemic and subsequent impact on therapists, research departments, and parents undoubtedly limited recruitment, yet the response rate for therapists was reasonable and covered all professional groups targeted. Non-response bias from high rates of partial questionnaire completion and selection bias towards more research-oriented therapists are concerns. Furthermore, despite our recruitment strategy targeting therapists in Wales, Scotland, and Northern Ireland by disseminating the survey through national professional bodies and networks, response rates from these devolved nations were very low. We used an evidence-based framework and related questionnaire for assessing acceptability, and the overall survey was systematically developed through a rigorous methodological process. However, most parents disengaged from responding after the first three questions, suggesting further involvement of parents in the questionnaire design may have improved the survey. Greater parental involvement in the overall study design may have also led to more diversity in our parent participants, e.g. inclusion of more fathers and participants with English as an additional language.

The low parental response rate in our survey could be due to factors related to the COVID-19 pandemic, the chosen research methods, and/or the questionnaire design, as the questionnaire was not tied to specific service user groups and was presenting a hypothetical, rather than specific, trial; however, the literature also suggests parents of children receiving therapy may feel uncertain about the concept of trials and participating in trials which needs to be considered as a potential factor related to parental (dis)engagement with our survey. A recent study exploring trial designs for children’s eating, drinking, and swallowing interventions [50] found that, whilst randomisation and indeed cluster randomisation was an acceptable feature of trials to parents, uncertainty about trial designs being appropriate and necessary for evaluating eating, drinking, and swallowing therapy interventions was expressed. Whilst the cluster randomised trial design was supported by parents in the context of eating, drinking, and swallowing interventions, case series studies were also put forward as a potential alternative. Goodwin et al. [45] in their study exploring the acceptability and feasibility of a specific trial of standing frames in children’s therapy found that less than half (43.2%, *n* = 32) of the parents from their survey would be willing to take part in a standing frames trial. Reasons cited included concerns about child factors (tolerance and pain) as well as perceptions of the risks and benefits of participation being a barrier to participation. With so few evaluation trials in children’s therapy and the field of childhood disability more broadly, there remains uncertainty about parent and child engagement in trials. There is a need to further explore how parent views and attitudes towards trials might affect recruitment and retention to future non-pharmacological intervention therapy trials and what strategies and supports might be beneficial to facilitate parent and child participation.

Whilst recognising the challenge to children’s therapy trials, it is also important to note that large-scale children’s therapy trials have been successfully conducted across several contexts, such as school-based practice [29, 51, 52] and sensory integration [3]. Their success suggests trials are possible in children’s therapy at least some of the time. More evidence is needed to explore the situations in which trials can be successfully conducted, how trial designs can be adapted to support their use, and what strategies facilitate trial delivery in the community context.

## Conclusions

This study found that, in principle, trials have coherence with children’s therapy, outcomes, and front-line care, both from the perspective of therapists and parents. However, therapists also reported varied levels of confidence in participating in a trial, high perceptions of burden associated with a trial, uncertainty of intention to do so, and potentially important negative and positive feelings related to participation. A range of factors that positively and negatively affect therapists’ perceptions about a trial were identified—including the potentially positive impact of appropriate support and resources, confidence with intervention allocation, and therapists’ sense of control and professional autonomy over their practice.

## Data Availability

The datasets generated and/or analysed during the current study are available in the Newcastle University repository (10.25405/data.ncl.17087333.v1).

## Abbreviations

A-Level: Advanced level
AAC: Augmentative and alternative communication
CHERRIES: Checklist for Reporting Results of Internet E-Surveys
CRCTs: Cluster randomised controlled trials
CRN: Clinical Research Network
GCE: General Certificate of Education
GCSE: General Certificate of Secondary Education
HRA: Health Research Authority
HSC: Health and Social Care
IQR: Interquartile range
ISRCTN: International Standard Randomised Controlled Trials Number
NHS: National Health Service
NIHR: National Institute for Health Research
O-level: Ordinary level
REC: Research Ethics Committee
RCTs: Randomised controlled trials
SPSS: Statistical Package for Social Sciences
TACT: Target, actions, context, time
TFA: Theoretical framework of acceptability
UK: United Kingdom
